# Lower serum potassium associated with increased mortality in dialysis patients: A nationwide prospective observational cohort study in Korea

**DOI:** 10.1371/journal.pone.0171842

**Published:** 2017-03-06

**Authors:** Sunhwa Lee, Eunjeong Kang, Kyung Don Yoo, Yunhee Choi, Dong Ki Kim, Kwon Wook Joo, Seung Hee Yang, Yong-Lim Kim, Shin-Wook Kang, Chul Woo Yang, Nam Ho Kim, Yon Su Kim, Hajeong Lee

**Affiliations:** 1 Department of Internal Medicine, Seoul National University Hospital, Seoul, Republic of Korea; 2 Department of Internal Medicine, Dongguk University Gyeongju Hospital, Gyeongju, Republic of Korea; 3 Seoul National University Hospital, Medical Research Collaborating Center, Seoul, Republic of Korea; 4 Seoul National University Kidney Research Institute, Seoul, Republic of Korea; 5 Department of Internal Medicine, Kyungpook National University Hospital, Daegu, Republic of Korea; 6 Department of Internal Medicine, Yonsei University College of Medicine, Seoul, Republic of Korea; 7 Department of Internal Medicine, Seoul St. Mary's Hospital, Seoul, Republic of Korea; 8 Department of Internal Medicine, Chonnam National University Hospital, Gwangju, Republic of Korea; The University of Tokyo, JAPAN

## Abstract

**Background:**

Abnormal serum potassium concentration has been suggested as a risk factor for mortality in patients undergoing dialysis patients. We investigated the impact of serum potassium levels on survival according to dialysis modality.

**Methods:**

A nationwide, prospective, observational cohort study for end stage renal disease patients has been ongoing in Korea since August 2008. Our analysis included patients whose records contained data regarding serum potassium levels. The relationship between serum potassium and mortality was analyzed using competing risk regression.

**Results:**

A total of 3,230 patients undergoing hemodialysis (HD, 64.3%) or peritoneal dialysis (PD, 35.7%) were included. The serum potassium level was significantly lower (*P* < 0.001) in PD (median, 4.5 mmol/L; interquartile range, 4.0–4.9 mmol/L) than in HD patients (median, 4.9 mmol/L; interquartile range, 4.5–5.4 mmol/L). During 4.4 ± 1.7 years of follow-up, 751 patients (23.3%) died, mainly from cardiovascular events (n = 179) and infection (n = 120). In overall, lower serum potassium level less than 4.5 mmol/L was an independent risk factor for mortality after adjusting for age, comorbidities, and nutritional status (sub-distribution hazard ratio, 1.30; 95% confidence interval 1.10–1.53; *P* = 0.002). HD patients showed a U-shaped survival pattern, suggesting that both lower and higher potassium levels were deleterious, although insignificant. However, in PD patients, only lower serum potassium level (<4.5 mmol/L) was an independent predictor of mortality (sub-distribution hazard ratio, 1.35; 95% confidence interval 1.00–1.80; *P* = 0.048).

**Conclusion:**

Lower serum potassium levels (<4.5 mmol/L) occur more commonly in PD than in HD patients. It represents an independent predictor of survival in overall dialysis, especially in PD patients. Therefore, management of dialysis patients should focus especially on reducing the risk of hypokalemia, not only that of hyperkalemia.

## Introduction

Patients with renal impairment have a high risk of potassium imbalance. Dyskalemia, both hyperkalemia and hypokalemia, can be responsible for the high prevalence of cardiovascular disease and even sudden death in dialysis patients by causing life-threatening cardiac arrhythmias. In the 4D trial, a study of diabetic patients undergoing hemodialysis (HD), the most common cause of death was not myocardial infarction, but sudden cardiac death, secondary to fatal arrhythmias [1].

While end-stage renal disease (ESRD) patients are prone to potassium imbalances, the presence of dyskalemia depends on the dialysis modality. In HD patients, most previous reports have demonstrated that hyperkalemia is relatively common and fatal [2, 3]. The Dialysis Outcomes and Practice Patterns Study database revealed that 6.3–20.0% of HD patients had serum potassium levels of more than 6 mmol/L at pre-dialysis [4]. This wide range can be attributed to ethnic differences. Likewise, the association between serum potassium and mortality also varies according to patient ethnicity [2, 5, 6]. In the Caucasian and African-American populations, hyperkalemia was associated with higher all-cause and cardiovascular mortality [2, 5], whereas in the Chinese population, hypokalemia was associated with elevated mortality in patients undergoing maintenance HD [6]. Different from HD patients, patients undergoing peritoneal dialysis (PD) are more commonly hypokalemic [7–11]. Furthermore, there is a recognized association between hypokalemia and an elevated risk of death [7, 8] and peritonitis [8]. A recent study showed lower serum potassium level is related to infectious-caused mortality in PD patients [12].

To date, studies investigating serum potassium levels and mortality according to dialysis modality had some limitations such as retrospective design [2, 7, 13], single-center cohort [13], or reduced sample size [6, 8]. The current report represents a prospective, multicenter, observational cohort study, where we extensively explored the association between serum potassium and mortality according to the modality of dialysis.

## Materials and methods

### Study participants

This investigation was based on a prospective, multicenter, cohort study using the database maintained by the Clinical Research Center for End Stage Renal Disease patients (CRC-ESRD) in Korea. The nationwide multicenter cohort was initiated for the purpose of improving survival rates and quality of life in ESRD patients. The CRC-ESRD cohort website incorporates all clinical outcomes and information from each center including cause of death and laboratory data. All participants provided their written consent to participate voluntarily in this cohort. This investigation was registered as a clinical trial (NCT00931970). Seoul National University Hospital Institutional Review Board approved with IRB number H-0905-047-281 between June 2009 and April 2015 and with IRB number H-1606-098-771 between May 2016 and June 2017.

Between August 2008 and October 2013, a total of 3,230 adult (age > 20 years) patients with ESRD, from over 31 hospitals and clinics across Korea, were registered in the CRC-ESRD cohort with informed consent; there were 2,078 HD patients and 1,152 PD patients. The cohort included participants who had progressed to ESRD and started incident dialysis just before enrolment in the study (n = 1,591), as well as prevalent dialysis patients, who had already been on dialysis for more than 3 months previous to enrolment in the study (n = 1,639). The patients who changed dialysis modality after registration were excluded from our analysis.

### Selected variables

The clinical data was collected in the form of web-based medical questionnaires. Well-trained data coordinators filled the questionnaire items by reviewing medical records or by in-person interviews. Pre-existing comorbidity data were obtained to calculate the Modified Charlson Comorbidity Index (MCCI). Various dialysis-related factors were also recorded, such as ESRD cause and type, as well as duration of dialysis. The cause of the ESRD was classified into 4 broad categories: diabetes; hypertension; glomerulonephritis; and other, which included interstitial nephritis, hereditary disease, neoplasms, and miscellaneous conditions. Dialysis vintage was defined as the duration of time between the first day of dialysis and the day the patient was enrolled in the study. Serum potassium was measured at enrollment and at the 3-month visit, and subsequently every 6 months until year five. Blood tests were performed before dialysis for HD patients, and randomly for PD patients. The representative value of the serum potassium level was calculated as an arithmetic mean of the values recorded over the follow-up years, excluding baseline levels (at the time of enrollment). Moreover, the following parameters representing nutritional status were obtained: body mass index (BMI), subjective global assessment (SGA), serum albumin, hemoglobin, cholesterol, uric acid, alkaline phosphatase, and highly sensitive C-reactive protein (hs-CRP). BMI was calculated as the weight (kg) divided by the square of the height (m). SGA was used to evaluate the overall protein-energy nutritional status, and included 6 subjective assessments, with 3 based on the patient’s history (weight loss, incidence of anorexia, and incidence of vomiting) and 3 based on the physician’s grading (muscle wasting, presence of edema, and loss of subcutaneous fat). Based on these assessments, each patient was given an SGA score of 1 to 7, as an indication of severe malnutrition, mild-moderate malnutrition, or normal nutritional status. Malnutrition was defined as an SGA score of 1 to 5.

### Outcome measurement

The specific cause and date of death for each patient registered in the CRC-ESRD cohort was retrieved from the medical record of the managing hospital and from the Korean National Statistical Office database. Data were recorded until December 2015. All cause-of-death data were recorded with one of the following six categories: cardiovascular, infectious, gastrointestinal, metabolic, other, and unknown. Cardiovascular mortality included myocardial infarction/ischemia, congestive heart failure, pulmonary edema, sudden cardiac death, and cerebrovascular disorder.

### Statistical analysis

To explore the association between serum potassium and mortality in dialysis patients, we fit a restricted cubic spline function. In addition, we stratified patients into subgroups based on the serum potassium levels (K) into 5 categories: K < 3.5 mmol/L; 3.5 ≤ K < 4.0 mmol/L; 4.0 ≤ K < 4.5 mmol/L; 4.5 ≤ K < 5.0 mmol/L; and K ≥ 5.0 mmol/L. Cumulative incidence curve was compared for each adjacent potassium-based subgroups using Gray’s test.

Fine and Gray’s regression analyses were used to evaluate the effect of serum potassium levels on mortality in both HD and PD patients. Variables that showed significant associations (*P* < 0.05) in the univariate Cox regression analysis or were of considerable theoretical relevance were entered into the multivariable Cox regression analysis in a step-wise manner. Model 1 was only adjusted by demographic variables such as age and sex. Model 2 was adjusted by comorbidity status such as MCCI score, in addition to Model 1 variables. Model 3 was additionally adjusted by nutritional and inflammatory markers such as BMI, SGA, serum albumin, alkaline phosphatase, uric acid, phosphorous and calcium. In case of hemodialysis patient, dialysis time per week and Kt/V were also included as adjusting variables in model 3.

Statistical analyses were performed using SAS 9.4 (SAS Institute, Cary, NC) and SPSS 18 (SPSS, Chicago, IL, USA), and statistical significance was defined as a *P*-value less than 0.05.

## Results

### Patient characteristics

**Fig 1:**
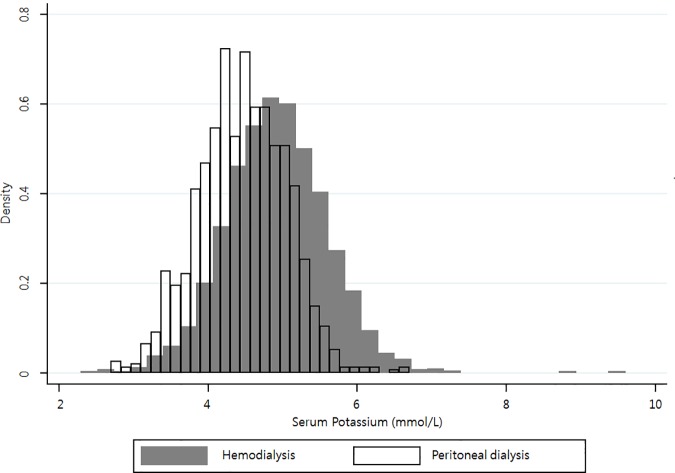
Distribution of serum potassium levels according to dialysis modality. The distribution is left-shifted in patients undergoing peritoneal dialysis.

A total of 3,230 patients were included in the analysis, comprising 2,078 HD patients and 1,152 PD patients. Table 1 shows the demographic, clinical, and laboratory characteristics of our patients at baseline (enrolment in the CRC-ESRD). The distribution of serum potassium levels differed according to the dialysis modality employed (Fig 1). In PD patients, the overall distribution of serum potassium levels was shifted to the left, and the mean level was significantly lower than that of HD patients (PD: 4.5 ± 0.6 mmol/L; HD: 4.9 ± 0.7 mmol/L; *P* < 0.001).

**Table 1:** Demographic and clinical characteristics of end-stage renal disease patients according to dialysis modality.

Variables	Total (N = 3,230)		*P*-value
	HD (n = 2,078)	PD (n = 1,152)	
Age (years)	59 [49–69]	54 [45–64]	<0.001^†^
Men (N, %)	1248 (60.1%)	651 (56.5%)	0.052^‡^
BMI (kg/m^2^)	22 [21–25]	23 [21–25]	<0.001^†^
SBP (mmHg)	141 [125–157]	135 [120–149]	<0.001^†^
DBP (mmHg)	75 [67–83]	80 [70–88]	0.003^†^
Dialysis vintage of prevalent patients (years)	2.92 [1.32–6.18]	3.21 [1.38–6.08]	<0.001^†^
Incidental dialysis (N, %)	1,142 (55.0%)	449 (39.0%)	<0.001^‡^
Primary renal disease (N, %)			<0.001^‡^
Diabetes	989 (47.6%)	401 (34.8%)	
Hypertension	323 (15.5%)	225 (19.5%)	
Glomerulonephritis	233 (11.2%)	185 (16.1%)	
Others	533 (25.6%)	341 (29.6%)	
MCCI	6 [4–7]	4 [3–6]	<0.001^†^
> High school (N, %)	1,259 (63.5%)	673 (67.2%)	0.049^‡^
Ever-smoker (N, %)	826 (39.7%)	379 (32.9%)	<0.001^‡^
Laboratory data			
Potassium (mmol/L)	4.9 [4.5–5.3]	4.5 [4.1–4.9]	<0.001^†^
Hemoglobin (g/dL)	9 [8–11]	10 [9–11]	<0.001^†^
Albumin (g/dL)	3.7 [3.2–4.0]	3.6 [3.3–3.9]	0.151^†^
Uric acid (mg/dL)	7.5 [6.2–8.9]	7.0 [6.0–8.5]	<0.001^†^
Alkaline phosphatase (IU/L)	80 [61–112]	102 [70–180]	<0.001^†^
Total cholesterol (mg/dL)	155 [125–179]	166 [141–198]	<0.001^†^
hs-CRP (mg/dL)	0.26 [0.04–1.42]	0.17 [0.03–0.86]	<0.001^†^
SGA (malnourished)	521 (28.1%)	186 (21.4%)	0.001^‡^
Medication (N, %)			
ARB or ACEI	575 (57.2%)	247 (60.1%)	0.314^‡^
Diuretics	551 (54.9%)	232 (56.4%)	0.638^‡^
Residual renal function (ml/min/1.73m^2^)	8.5 [4.4–15.2]	7.0 [3.7–14.3]	0.032^†^

Abbreviation: HD, hemodialysis; PD, peritoneal dialysis; BMI, body mass index; SBP, systolic blood pressure; DBP, diastolic blood pressure; MCCI, modified Charlson comorbidity index; SGA, subjective global assessment; ARB, angiotensin receptor blocker; ACEI, angiotensin converting enzyme inhibitor

^†^ Mann-Whitney U test was done for continuous nonparametric variables with interquartile range in square brackets.

^‡^ Chi-square test for categorical nonparametric variables.

### Comparison of characteristics according to serum potassium levels

Next, we compared baseline characteristics between the subgroups defined according to serum potassium levels (Table 2). Compared to patients with higher serum potassium levels, those with lower serum potassium levels were older, had more comorbidities (expressed in terms of MCCI), and showed lower serum albumin and serum uric acid levels. However, there was no difference among the subgroups defined according to serum potassium levels regarding BMI, cholesterol levels, or proportion of patients with malnutrition (evaluated in terms of SGA). The proportion of PD patients increased as serum potassium levels decreased (*P* < 0.001). Interestingly, patients with lower serum potassium levels showed higher crude mortality rate (*P* < 0.001), in spite of the shorter follow-up duration (*P* = 0.001) and shorter dialysis vintage (*P* = 0.044).

**Table 2:** Baseline characteristics of end-stage renal disease patients stratified according to serum potassium levels (N = 3,230).

Variables	K<3.5 (n = 91)	3.5≤ K <4.0 (n = 289)	4.0≤ K <4.5 (n = 717)	4.5≤ K <5.0 (n = 962)	K ≥5 (n = 1,171)	*P*-value
Age (years)	70.0 [54.0–73.0]	63.0 [51.0–73.5]	63.0 [51.0–72.0]	60.0 [49.3–69.0]	59.0 [50.0–68.0]	<0.001^†^
Men (N, %)	51 (56.0%)	157 (54.3%)	419 (58.4%)	590 (61.3%)	682 (58.2%)	0.248^‡^
BMI (kg/m^2^)	22.0 [21.0–24.0]	22.0 [20.0–25.0]	23.0 [21.0–24.0]	23.0 [21.0–25.0]	23.0 [21.0–25.0]	0.163^†^
SBP (mmHg)	135 [124–149]	133 [121–150]	140 [122–152]	140 [120–154]	140 [127–156]	<0.001^†^
DBP (mmHg)	72 [65–86]	78 [67–89]	77 [65–86]	78 [69–84]	79 [70–86]	<0.001^†^
Dialysis vintage (years)	2.26 [0.65–6.53]	2.81 [1.17–6.34]	2.75 [1.25–5.63]	2.87 [1.30–5.56]	3.35 [1.50–6.81]	0.044^†^
Incident dialysis (N, %)	42 (46.2%)	155 (53.6%)	381 (53.1%)	500 (52.0%)	513 (43.8%)	<0.001^‡^
Dialysis modality (HD, %)	29 (31.9%)	108 (37.4%)	378 (52.7%)	621 (64.6%)	942 (80.4%)	<0.001^‡^
Primary renal disease (N, %)						0.068^‡^
Diabetes	39 (42.9%)	116 (40.1%)	291 (40.6%)	434 (45.1%)	510 (43.6%)	
Hypertension	10 (11.0%)	47 (16.3%)	139 (19.4%)	162 (16.8%)	190 (16.2%)	
Glomerulonephritis	8 (8.8%)	30 (10.4%)	96 (13.4%)	127 (13.2%)	157 (13.4%)	
Others	34 (37.4%)	96 (33.2%)	191 (26.6%)	239 (24.8%)	314 (26.8%)	
MCCI	6.0 [4.0–8.0]	6.0 [4.0–8.0]	5.0 [3.0–7.0]	5.0 [3.0–7.0]	5.0 [3.0–7.0]	0.019^†^
> High school (N, %)	57 (62.6%)	172 (59.5%)	414 (57.7%)	625 (65.0%)	755 (64.5%)	0.014^‡^
Smoking experience (N, %)	30 (34.5%)	97 (36.5%)	275 (39.4%)	374 (40.0%)	429 (38.2%)	0.700^‡^
Laboratory data						
Potassium (mmol/L)	3.3 [3.1–3.4]	3.8 [3.6–3.9]	4.3 [4.2–4.4]	4.7 [4.6–4.9]	5.3 [5.1–5.6]	<0.001^†^
Hemoglobin (g/dL)	9.0 [8.0–11.0]	10.0 [9.0–11.0]	10.0 [9.0–11.0]	10.0 [8.0–11.0]	10.0 [8.0–11.0]	0.693^†^
Albumin (g/dL)	3.0 [3.0–4.0]	4.0 [3.0–4.0]	4.0 [3.0–4.0]	4.0 [3.0–4.0]	4.0 [3.0–4.0]	<0.001^†^
Alkaline phosphatase (IU/L)	80 [50–121]	106 [62–199]	95 [69–154]	89 [61–128]	83 [64–117]	0.026^†^
Uric acid (mg/dL)	7.0 [5.0–9.0]	7.0 [6.0–8.0]	7.0 [6.0–9.0]	8.0 [6.0–9.0]	8.0 [7.0–9.0]	<0.001^†^
Total cholesterol (mg/dL)	147 [117–194]	155 [130–194]	156 [129–191]	154 [127–183]	157 [134–189]	0.084^†^
hs-CRP (mg/dL)	0.0 [0.0–2.0]	0.0 [0.0–1.0]	0.0 [0.0–1.0]	0.0 [0.0–1.0]	0.0 [0.0–1.0]	<0.001^†^
Medication (N, %)						
ARB or ACEI	17 (48.6%)	74 (57.4%)	197 (59.0%)	247 (55.0%)	287 (61.1%)	0.299^‡^
Diuretics	17 (48.6%)	79 (61.2%)	196 (58.7%)	234 (52.3%)	257 (54.8%)	0.227^‡^
Residual renal function	6.8 [4.1–12.5]	6.1 [3.7–13.3]	8.8 [4.9–15.0]	8.9 [4.7–15.2]	8.0 [4.1–15.2]	0.276^†^
SGA (malnourished, N, %)	24 (26.4%)	67 (23.2%)	174 (24.3%)	204 (21.2%)	238 (20.3%)	0.226^‡^
Death	42 (46.2%)	95 (32.9%)	197 (27.5%)	206 (21.4%)	211 (18.0%)	<0.001^‡^
Follow up duration (years)	3.5 [3.1–4.0]	4.0 [3.8–4.2]	4.2 [4.1–4.3]	4.5 [4.4–4.6]	4.6 [4.5–4.7]	<0.001^†^

Abbreviations: BMI, body mass index; SBP, systolic blood pressure; DBP, diastolic blood pressure; HD, hemodialysis; MCCI, modified Charlson comorbidity index; ARB, angiotensin receptor blocker; ACEI, angiotensin converting enzyme inhibitor; SGA, subjective global assessment.

^†^ Kruskal Wallis test was applied for continuous nonparametric variables.

^‡^ The Chi-square test was applied for categorical nonparametric variables.

### Serum potassium and mortality according to dialysis modality

**Fig 2:**
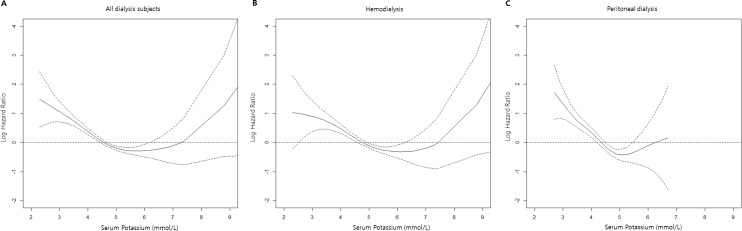
Log hazard ratio for overall mortality in relation to serum potassium in patients with end-stage renal disease. A) entire study cohort, B) patients undergoing hemodialysis, C) patients undergoing peritoneal dialysis. The solid line and dotted lines represent the central risk estimate and 95% confidence intervals, respectively.

During 4.4 ± 1.7 years of follow-up period, 23.3% of the patients (751 of 3,230) died, including 22.9% of the HD patients (476 of 2,078) and 23.9% of the PD patients (275 of 1,152). Fig 2 displays the restricted cubic spline plot that graphically describes the association between serum potassium and overall mortality in the entire study cohort, and in the patient groups defined by dialysis modality. A U shaped curve was found for both the entire study cohort (Fig 2A) and the HD group (Fig 2B), although, in both cases, the right arm of the curve increased dramatically for serum potassium levels >7.5 mmol/L. On the other hand, the left arm for PD patients showed a significant incline of overall mortality with below 4.5 mmol/L (Fig 2C).

**Fig 3:**
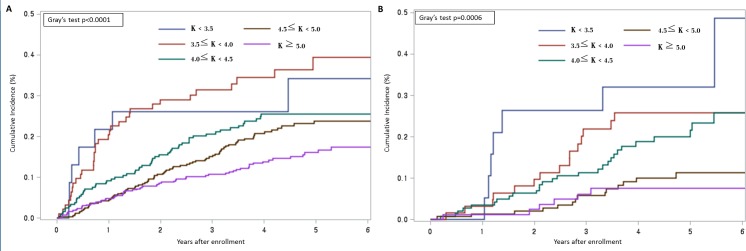
**Cumulative mortality incidence in patients with end-stage renal disease undergoing hemodialysis (A) or peritoneal dialysis (B).** Mortality incidence was plotted according to the level of serum potassium.

Fig 3 shows the cumulative mortality incidence according to dialysis modality and serum potassium level (K). In both HD and PD patients, there was a significant difference in survival probability among the 5 potassium-based groups (Gray’s test, *P* < 0.001). Lower serum potassium groups show a tendency of higher mortality incidence in both groups. Of note, PD patients showed a significant difference in mortality rate for serum potassium at the level of 3.5 mmol/L and 4.5 mmol/L.

### Survival analysis according to serum potassium level

To explore the effect of serum potassium on all-cause mortality, we performed Fine and Gray’s regression analyses. Table 3 summarizes the univariate and multivariable Fine and Gray’s regression models according to dialysis modality. In the entire study cohort, serum potassium <4.5 mmol/L was proven to be a significant risk factor for mortality (unadjusted SHR, 1.71; 95% CI, 1.48–1.97; *P* < 0.001), compared with serum potassium ≥ 4.5 mmol/L. Even after adjustment of covariables, serum potassium levels <4.5 mmol/L remained an independent risk factor for mortality (adjusted SHR, 1.30; 95% CI, 1.10–1.53; *P* < 0.001). This relationship was manifested similarly in PD patients (adjusted SHR, 1.35; 95% CI, 1.00–1.80; *P* = 0.048), but not in HD patients.

**Table 3:** Competing risk regression analyses of mortality in patients with serum potassium < 4.5 mmol/L according to dialysis modality.

Model (Number of mortality cases)	SHR (95% CI)	*P*-value
Total		
Unadjusted (751)	1.71 (1.48, 1.97)	<0.001
Model I (751)	1.58 (1.37, 1.82)	<0.001
Model II (741)	1.55 (1.34, 1.79)	<0.001
Model III (644)	1.30 (1.10, 1.53)	0.002
Hemodialysis		
Unadjusted (476)	1.64 (1.35, 1.99)	<0.001
Model I (476)	1.39 (1.15, 1.68)	<0.001
Model II (469)	1.30 (1.07, 1.58)	0.009
Model III (327)	1.14 (0.88, 1.46)	0.319
Peritoneal dialysis		
Unadjusted (275)	2.06 (1.61, 2.64)	<0.001
Model I (275)	1.60 (1.25, 2.05)	<0.001
Model II (272)	1.56 (1.21, 2.01)	<0.001
Model III (215)	1.35 (1.00, 1.80)	0.048

Participants with serum potassium levels ≥ 4.5 mmol/L were considered as the reference group. Values given as hazard ratio (95% confidence interval).

Abbreviations: K, serum potassium levels; SHR, Sub-distribution hazard ratio; CI, confidence interval; MCCI, modified Charlson comorbidity index; BMI, body mass index; SGA, subjective global assessment; ALP, alkaline phosphatase.

Model I: adjusted for age and sex.

Model II: adjusted for Model I parameters as well as for MCCI.

Model III: adjusted for model II parameters as well as for BMI, SGA, albumin, ALP, Uric acid, Phosphorous, and Calcium (including dialysis time and Kt/V for hemodialysis).

**Fig 4:**
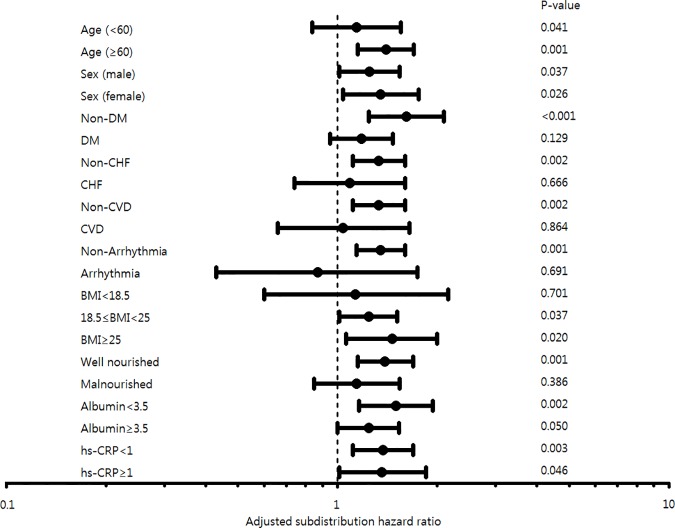
Forest plot representing log hazard ratios for mortality risk in the entire study cohort patients with serum potassium < 4.5 mmol/L. Results assessed according to known or potential risk factors. Abbreviations: DM, diabetes mellitus; CHF, congestive heart failure; CVD, cardiovascular disease; BMI, body mass index; hs-CRP, high sensitivity C-reactive protein.

Because this association between lower serum potassium levels and higher mortality might be affected by nutritional or inflammatory status, we performed the same analysis dissected by age, sex, presence of comorbidities, BMI, SGA, serum albumin, and hs-CRP (Fig 4). Serum potassium levels below 4.5 mmol/L were associated with elevated mortality especially in older (age ≥ 60 years), BMI ≥ 18.5 kg/m^2^ and well-nourished patients (SGA 6–7). In addition, lower serum potassium levels had an impact on mortality rate in patients without comorbidities such as diabetes, congestive heart failure, cerebrovascular disease, and arrhythmia. Notably, lower serum potassium levels influenced mortality rates irrespective of the level of serum albumin and hs-CRP.

### Causes of death

**Fig 5:**
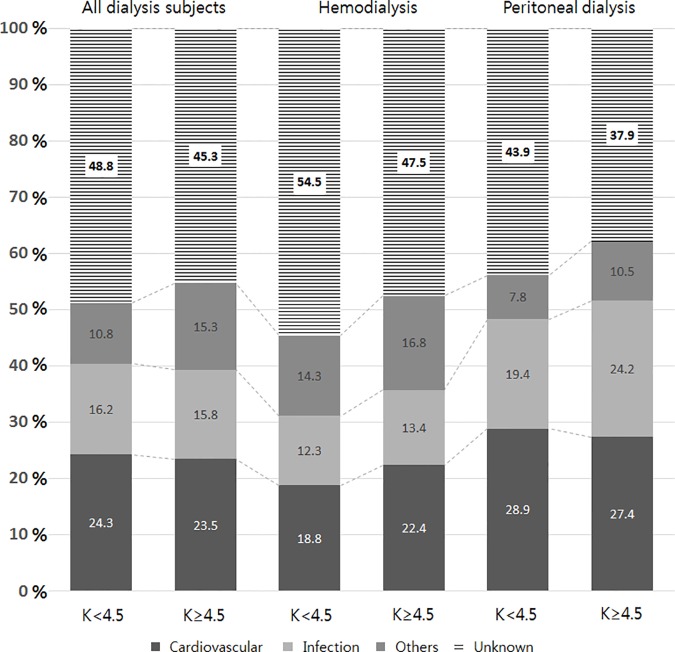
The distribution of causes of death in the study patients. In the entire study cohort, the distribution of causes of death was similar for serum potassium levels below and above 4.5 mmol/L. However, subsequent analysis established that patients undergoing hemodialysis and those undergoing peritoneal dialysis show slightly different distribution of causes of death. Specifically, hemodialysis patients with serum potassium ≥4.5 mmol/L were more likely to die of cardiovascular disease than those with serum potassium < 4.5 mmol/L. On the other hand, peritoneal dialysis patients with serum potassium ≥4.5 mmol/L displayed a tendency to die of infection than those with serum potassium < 4.5 mmol/L, although insignificant.

The cause of death was known only for 440 of the 751 death events recorded. Overall, cardiovascular disease (24.4%) and infection (15.3%) were the major causes of death. The distribution of causes of death was not different according to serum potassium levels, as shown in Fig 5. Because of lots of missing data of cause of death, it was hard to observe any significance between causes of death and serum potassium groups.

## Discussion

The results of our current study suggest that hypokalemia is more commonly found in PD patients than in HD patients; moreover, lower serum potassium (< 4.5 mmol/L) is an independent risk factor for mortality in dialysis patients, and this phenomenon is more pronounced for PD patients; finally, the major cause of death in PD patients with lower potassium was cardiovascular death and infection.

Lower potassium levels have been suggested as a surrogate marker of malnutrition, possibly related to increased mortality [14–17]. Malnourished subjects who are identified as having malnutrition, inflammation, and atherosclerosis (MIA syndrome) are common among ESRD patients; MIA syndrome is also known to be related to increased mortality [17–20]. We may, therefore, suppose that hypokalemia is associated with higher mortality rates due to a lack of nourishment. However, even after adjusting for nutritional factors such as SGA and BMI, lower serum potassium level was still an independent risk factor for mortality in the subgroup analysis.

The naturally emerging question here is: why do PD patients have lower potassium serum levels, and why does lower potassium level itself increase the mortality risk? We can consider that lower serum potassium is induced by glucose absorption from dialysate, resulting in the intracellular uptake of potassium accompanied by insulin release [21]. A muscle biopsy study found increased muscle potassium content in continuous ambulatory peritoneal dialysis patients, supporting this hypothesis [22, 23]. Moreover, residual renal function is much better preserved in PD than HD [24–27]. The ability to excrete urinary potassium is retained to a higher degree in PD patients.

Moreover, most peritoneal dialysate is a potassium-free solution, whereas the majority of HD patients use a dialysate potassium bath containing a potassium concentration in the range of 1.1 to 2.0 mmol/L [2]. In terms of potassium intake, Asian individuals tend to have a lower potassium intake, expressed in terms of the potassium levels in the urine collected over a period of 24 hours: South Korea, 47.5 mmol/day; China, 33.9 mmol/day; Japan, 40.7 mmol/day; Taiwan 29.8 mmol/day. For comparison, potassium intake in individuals from Western countries was reported at: United States, 52.4 mmol/day; Italy 56.6 mmol/day; Germany 70.7 mmol/day; United Kingdom, 56.6 mmol/day [28]. These facts can help to explain why PD patients, especially Asian patients, are more likely to experience a hypokalemic condition.

Hypokalemia has been known to play various physiological roles that may contribute to increasing mortality rates. First, there are two major cardiovascular side effects of hypokalemia: hypertension and ventricular arrhythmia. Several lines of evidence revealed that potassium deficiency can increase blood pressure, and this has been shown to be linked to endothelial dysfunction [29–32]. Therefore, oral potassium supplements have been recommended for the control of blood pressure [33]. Moreover, in patients with cardiovascular disease, hypokalemia is associated with arrhythmia and increased mortality [34–37]. The direct physiologic mechanism by which hypokalemia increases the risk of cardiovascular events has not yet been determined. However, substantial epidemiologic and clinical evidence supports this hypothesis. The second physiological role of hypokalemia, with respect to endocrinology, is the impairment of both insulin release and end-organ sensitivity to insulin, resulting in hyperglycemia [38–40]. The incidence of end-organ complications is known to be increased in diabetic patients with hyperglycemia. Controlling hypokalemia, therefore, may decrease adverse endocrinological effects. Third, hypokalemia can result in several muscle-related complications. Specifically, hypokalemia can impair the depolarization development in muscle cells, affecting muscle contraction [41]. Hypokalemia may also affect the smooth muscles of the gastrointestinal tract, leading to decreased appetite and even paralytic ileus [42], which is possibly related to malnutrition.

If lower serum potassium in PD patients has a deleterious effect on survival, efforts to correct serum potassium levels should be considered. A previous propensity-matched study involved an analysis of the effect of oral potassium-supplement use on overall mortality and hospitalization outcomes in patients with chronic heart failure [43]. Because, in such patients, hypokalemia has been known to be associated with increased mortality, the use of supplements was expected to improve outcomes. However, the use of potassium supplements was not found to be associated with reduced mortality, but with increased hospitalization due to cardiovascular causes and progressive heart failure.

In HD patients, there was a report that higher potassium intake is associated with increased mortality [44]. However, the mean value of serum potassium reported in the present study was 4.9 mmol/L. Therefore, the impact of dietary potassium supplements on hypokalemic HD patients could not be determined, as the patients were not prescribed such supplements. In PD patients, potassium supplementation via oral route or dialysate has been undertaken, in addition to rigorous dietary management [42, 45, 46]. Some clinical studies have shown that using glucose-free icodextrin dialysate instead of glucose-containing dialysate improved serum potassium levels and reduced the prevalence of hypokalemia [47, 48]. There have been several trials investigating the maintenance of serum potassium levels within the normal range. However, it remains unknown whether or not correction of hypokalemia improves survival in PD patients.

The strength of our study lies in the fact that all analyses were performed on data from a large, prospective, nationwide, cohort study, which is regularly (twice a year) monitored using surveys to validate the quality of the data. Thus, we were able to confirm that lower serum potassium is an independent risk factor for mortality in PD patients. Moreover, we found that serum potassium levels do not influence mortality as much in HD patients as in PD patients.

Nevertheless, there are several limitations to be considered regarding the interpretation of the results. First, our study cohort included only 6 PD patients with K > 6.0 mmol/L, of whom only 2 died. Such low prevalence of hyperkalemia in PD patients was also observed in BRAZPD II study [12]. Therefore, there would be insufficient statistical significance in the claim that mortality is low in PD patients with hyperkalemia; the same observation leads to an indication to focus on hypokalemic PD patients, who are far more prevalent and exposed to higher mortality rate. Second, significantly more data regarding cause of death were missing for HD patients (n = 213, 44.7%) than for PD (n = 98, 35.6%). That is to say, it is not relevant to discuss the exact proportion of causes of death according to dialysis modality and serum potassium levels. Third, serum iPTH and magnesium levels were not included as adjusting variables in regression analysis though they have been known as independent risk factor for mortality [49–52]. Fourth, there can be controversial issues about selection bias not to include only incident patients but to include both incident and prevalent patients. However, we also observed the similar risk pattern with only incident cases. Another limitation is that laboratory tests were performed independently in each participating center. Therefore, our results should be interpreted in caution.

In summary, our study demonstrated the impact of serum potassium levels on survival, according to dialysis modality. Overall, lower serum potassium levels (<4.5 mmol/L) proved an independent risk factor for ESRD patients undergoing dialysis. HD patients showed a U-shaped, although insignificant, association between lower serum potassium levels and mortality, while PD patients revealed an apparent reverse association between serum potassium levels and mortality. In conclusion, the management of dialysis patients should be focused on reducing the risk of hypokalemia in addition to reducing that of hyperkalemia.

## Supporting information

S1 File Extracted raw data of CRC-ESRD cohort study. (ZIP)
